# CFD Modeling of Airflow and Aerosol Transport in the Human Respiratory System: A Comprehensive Review

**DOI:** 10.1007/s11095-026-04080-w

**Published:** 2026-05-04

**Authors:** Dogan Ciloglu, Hacer Ucuncu

**Affiliations:** Department of Mechanical Engineering, Atatürk University, Erzurum, Turkey

**Keywords:** aerosol particles, CFD, computational models, *in silico*, respiratory system

## Abstract

**Background:**

Accurate modelling of airflow and aerosol/particle dynamics within the human respiratory system is essential for improving inhalation-based drug delivery strategies and for evaluating the health risks associated with hazardous particulates. Owing to the complex geometry of the human airways, inter-individual anatomical variations, and variable breathing patterns, this process constitutes a highly complex multiphase flow problem. To address the constraints associated with *in vivo* and *in vitro* techniques, *in silico* approaches based on computational fluid dynamics (CFD) have been extensively utilized to examine respiratory airflow and aerosol dynamics at microscopic scales.

**Objectives:**

The aim of this study is to review recent CFD-based approaches for modeling airflow and aerosol behavior in the human respiratory system, summarize key modeling strategies and influential parameters, and identify future research directions.

**Results:**

Recent studies indicate a transition of respiratory tract models toward more physiologically realistic and whole-lung representations. These studies demonstrate that coupling CFD with particle models enables reliable prediction of aerosol transport and deposition by accounting for the effects of geometric variations, breathing conditions, turbulence characteristics, and particle physical and chemical properties.

**Conclusion:**

CFD-based modeling, particularly when integrated with particle dynamics, provides a powerful and reliable framework for investigating airflow and aerosol behavior in the human respiratory system. Continued advancements toward realistic whole-lung models and improved representation of physiological and particle-related parameters are expected to further enhance predictive accuracy and support both clinical and environmental health applications.

## Introduction

Airborne pollutants, other than therapeutically administered drug particles intended to reach the lungs via inhalation, pose serious threats to human health. Environmental pollution, particularly in developing countries, has led to a significant increase in respiratory diseases. Among these, chronic obstructive pulmonary disease (COPD), lung cancer, tuberculosis, pneumonia, asthma, pharyngitis, cystic fibrosis, emphysema, and the globally impactful COVID-19 syndrome in recent years stand out as major public health concerns.

According to the World Health Organization (WHO), COPD affects more than 300 million people worldwide and causes approximately 3.2 million deaths annually. While COPD is projected to become the third leading cause of death globally by 2030, it is reported to rank fourth in Turkey. Lung cancer likewise remains a major public health concern, particularly in industrialized countries; it accounted for 1.8 million deaths in 2018, and this number is expected to rise to 3 million by 2035. Moreover, tuberculosis and pneumonia continue to be significant sources of morbidity and mortality, notably in low- and middle-income countries, while asthma is a common chronic respiratory disease with an estimated global prevalence of 334 million. Finally, the COVID-19 pandemic, which has had a global impact since 2019, has once again brought to the forefront the effects of respiratory diseases and airborne particles on human health. Although the WHO declared the end of the global public health emergency in mid-2023, it continues to emphasize the persistence of risks associated with emerging variants and the importance of preventive strategies at the global scale. Collectively, these data underscore the critical importance of accurately modeling airflow and aerosol particle transport within the respiratory tract for elucidating disease mechanisms and for the development of effective preventive and therapeutic approaches.

During inhalation (inspiration), the thoracic cavity expands primarily as a result of the downward movement of the respiratory muscles, most notably the diaphragm. This expansion generates negative pressure within the thorax, allowing air to flow through the nose and mouth, pharynx, larynx, trachea, bronchi, and bronchioles, ultimately reaching the alveolar region. Gas exchange of O_2_ and CO_2_ takes place in the alveolar region of the lungs, which constitute the principal organs of the respiratory system. During exhalation (expiration), air follows the same pathway in the reverse direction and is expelled from the body; this cyclic process is defined as respiration. In addition to maintaining blood pH homeostasis and regulating body temperature, the primary function of the respiratory system is to meet the body’s oxygen demand through the lungs and the extensive airway network [1].

Inhaled medications are widely and effectively used in the treatment of respiratory diseases due to their rapid onset of action, prolonged therapeutic effects, and relatively low incidence of systemic side effects. By delivering aerosolized drugs to the lungs through the nasal or oral airways at low doses, this approach provides a convenient and widely applicable treatment option for patients. In such therapies, several parameters influence drug delivery, including micro/nano-particle formulation, particle deposition within the respiratory tract, inhaler device characteristics, and exposure to toxic aerosols [2]. On the other hand, the success of inhalation therapy is strongly dependent on disease severity; impaired lung function and hyperinflation may prevent adequate inspiration, thereby reducing therapeutic efficacy. Consequently, accurate dose determination, along with precise characterization of particle deposition and transport within the airways, is of paramount importance for treatment success. A realistic and efficient characterization of aerosol dynamics along the human respiratory tract plays a critical role in both assessing toxic particle deposition and developing effective treatments for a wide variety of lung diseases.

In *vivo* studies involve experimental tests conducted on human and animal subjects to determine the distribution of radiolabeled aerosols within the respiratory system. Although they provide the most accurate and meaningful reference datasets for other lines of research, these approaches are accompanied by substantial limitations, including high imaging costs, lengthy labeling procedures, risks associated with radiation exposure, technical complexity, and ethical constraints-particularly when human subjects are involved. Moreover, variations in inhalation device operating principles and individual anatomical differences further complicate data analysis and the interpretation of underlying mechanisms [3].

In contrast, *in vitro* investigations examine aerosol transport and deposition through experimental studies employing simplified apparatuses or physical models of the human respiratory tract [4–6]. This approach offers several advantages, such as lower cost, ease of implementation, absence of ethical concerns, and precise control of aerosol/fluid conditions. However, despite recent advances, significant limitations remain in accurately representing aerosol deposition in the peripheral airways, primarily because most *in vitro* studies focus on the upper respiratory tract and existing models often fail to capture the complex airway anatomy and dynamic flow conditions with sufficient resolution.

Computational fluid dynamics (CFD) is an *in silico* modeling approach that enables the solution of transport equations by simulating airflow (laminar/turbulent) and particle dynamics (transport/deposition). Owing to the high cost and practical challenges associated with experimental methods, CFD simulations have been reliably employed for decades in a wide range of applications, from the design of pharmaceutical inhalers to the prediction of aerosol behavior within the respiratory tract, allowing for detailed modeling of drug and particle transport in 3D airway geometries [7, 8]. CFD analyses provide substantial support to healthcare professionals, particularly in the diagnosis and treatment planning of respiratory diseases such as COPD, by offering not only 3D distributions of pressure and velocity fields but also access to parameters that are difficult to obtain experimentally, including pressure gradients and wall shear stress. Furthermore, the resulting three-dimensional simulations may be regarded as potential clinical tools for the development of personalized, patient-specific treatment strategies.

In *in silico* studies, the flow field is solved using the Navier–Stokes equations in conjunction with turbulence and heat/mass transfer models [9]. Particle motion is modeled based on Newton’s second law, accounting for forces such as drag, gravity, Brownian motion, and heat/mass transfer effects. Owing to the flexibility provided by CFD simulations, a broad spectrum of airway geometries, aerosol characteristics, and delivery systems can be analyzed without reliance on empirical deposition correlations, thereby supporting the development of novel inhaler designs and performance optimization studies [10, 11]. Recent advances in computational efficiency and reductions in simulation cost and time have substantially alleviated the limitations imposed by model complexity, leading to a growing number of studies focused on regional aerosol deposition [12, 13]. Nevertheless, the extent to which CFD predictions accurately represent local deposition patterns of inhaled aerosols remains uncertain, and such findings should be interpreted with caution until validated against high-resolution experimental data [14]. Therefore, enhancing the reliability of CFD models through complementary experimental studies is of critical importance for achieving a more accurate understanding of key parameters such as inhaler performance and aerosol deposition.

To date, numerous numerical studies have employed both simplified and anatomically realistic models of the respiratory tract. In particular, the use of realistic geometries has enabled aerosol dynamics within the human respiratory system to be simulated with a high degree of physiological fidelity. Such models have made significant contributions by allowing the identification of where and to what extent particulate matter deposits within the airways, the analysis of airflow velocities under different breathing conditions, and the extraction of critical insights relevant to the treatment processes of respiratory diseases. With the incorporation of realistic airway geometries, particle deposition in constricted and geometrically complex regions, such as the trachea and bifurcation zones, can now be predicted with greater accuracy. Owing to the inherent limitations of *in vivo* data, researchers have generally evaluated these CFD-based predictions by comparison with *in vitro* measurements rather than direct *in vivo* datasets.

Over the past two decades, research efforts have substantially advanced the understanding of particle deposition and airway flow characteristics. In parallel with technological progress, image-based anatomical model development has gained considerable momentum. The objective of the present study is to comprehensively compile and critically evaluate the latest developments in computational modeling of airflow and particle deposition within the human respiratory system, with particular emphasis on developments in CFD-based modeling approaches. Within this framework, the study aims to elucidate the evolution of advanced models that incorporate detailed representations of airway geometry and physiological features, and to systematically examine the influence of key geometric and physiological parameters on respiratory airflow and particle deposition. By identifying the limitations of the existing literature, this work also provides recommendations to guide future research directions.

## The Human Respiratory System

**Fig. 1:**
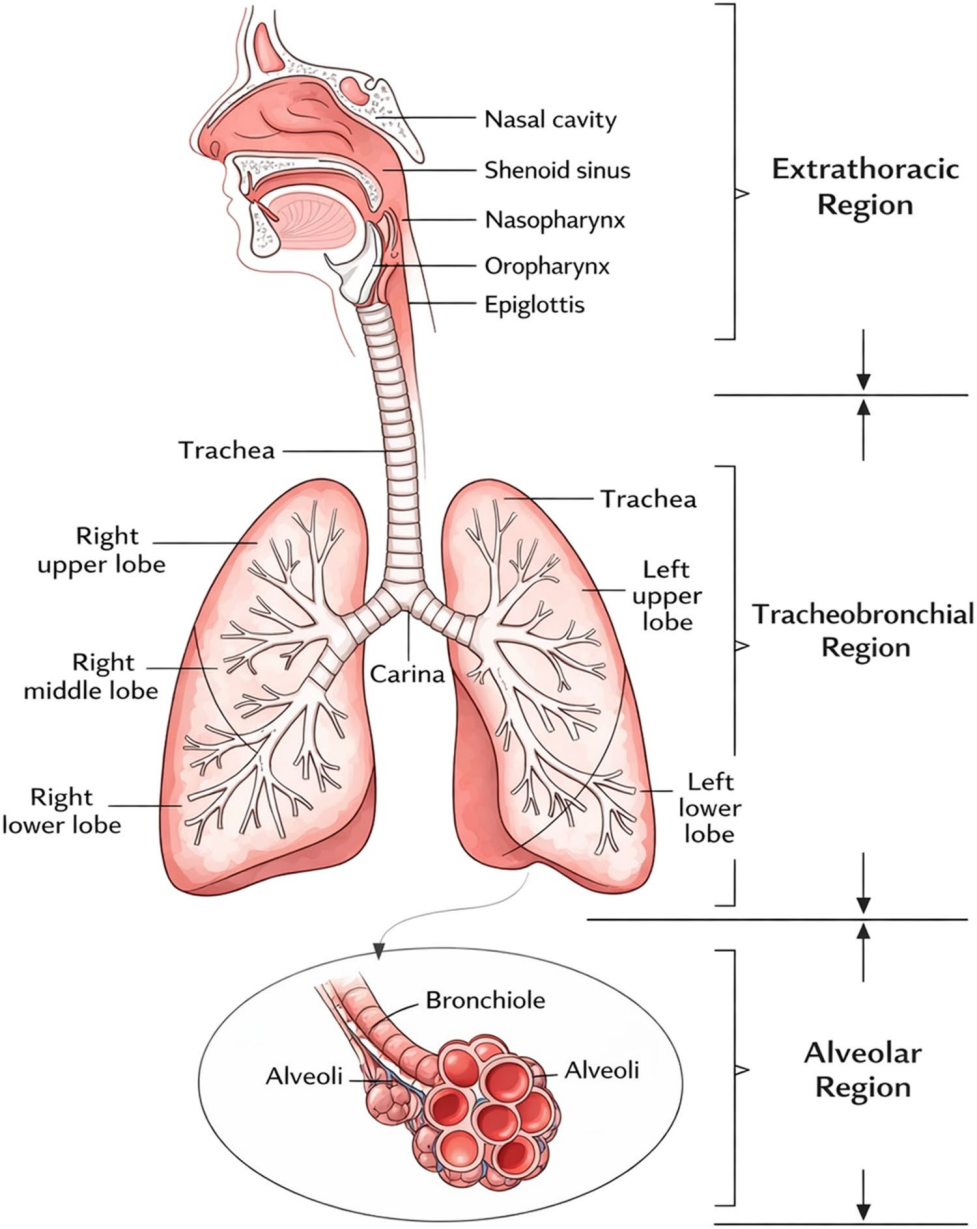
Schematic view of the human respiratory system (adapted from [15])

The human respiratory system exhibits a highly complex structure and is typically divided into three main regions: the extrathoracic (ET), tracheobronchial (TB), and alveolar regions (Fig. 1). The ET region, commonly referred to as the upper respiratory tract, comprises the mouth, nose, pharynx, and larynx. The pharynx is further subdivided into three sections based on its anatomical location: the oropharynx (posterior to the oral cavity), nasopharynx (posterior to the nasal cavities), and laryngopharynx (posterior portion of the pharynx). The epiglottis is a critical component of the larynx that prevents ingested food from entering the trachea by closing the airway during swallowing. The TB region constitutes the lower respiratory tract and consists of the trachea, bronchi, and the airway passages extending to the respiratory bronchioles, which conduct air into the lungs [15].

In this complex, tree-like geometry, the location of an individual airway is commonly identified by a corresponding generation number. According to Weibel’s model, the human respiratory system comprises 23 generations, with the first 16 generations (G0-G16) representing the TB region, which consists of the trachea and the branching air-conducting pathways, namely the bronchi and bronchioles, arranged in a tree-like structure [16]. Within this region, air is conveyed toward the alveolar compartment. The two primary airways branching from the trachea are referred to as the main bronchi. These bronchi supply two lobes in the left lung and three lobes in the right lung, respectively, before further branching into segmental bronchi. The cartilaginous rings present in the trachea and main bronchi result in a corrugated inner surface of these airways.

Beyond the TB region, the airways continue with the respiratory bronchioles, where alveolar sacs begin to appear, and terminate at the 23rd generation with fully developed alveolar sacs. This region, formed by the respiratory bronchioles, alveolar ducts (*ductus alveolaris*), and alveolar sacs (*saccus alveolaris*), is defined as the alveolar (*acinus*) region. The alveolar region corresponds to generations G17-G23 and is responsible for gas exchange.

## Airway Models

Prior to the development of detailed computational and image-based airway geometries, regional aerosol deposition was frequently estimated using simplified semi-empirical and compartmental airway representations. Frameworks such as the National Council on Radiation Protection and Measurements (NCRP) model provided rapid predictions of total and regional deposition fractions based on experimentally derived correlations and idealized airway structures [17]. These approaches offered low computational cost and practical applicability, particularly in inhalation toxicology and radiation protection studies. However, their simplified one-dimensional or compartment-based formulations lack the spatial resolution required to capture local flow structures, heterogeneous deposition patterns, and subject-specific anatomical variability. Consequently, more detailed geometric and CFD-based airway models have increasingly replaced these methods for high-fidelity prediction of airflow and particle transport.

**Fig. 2:**
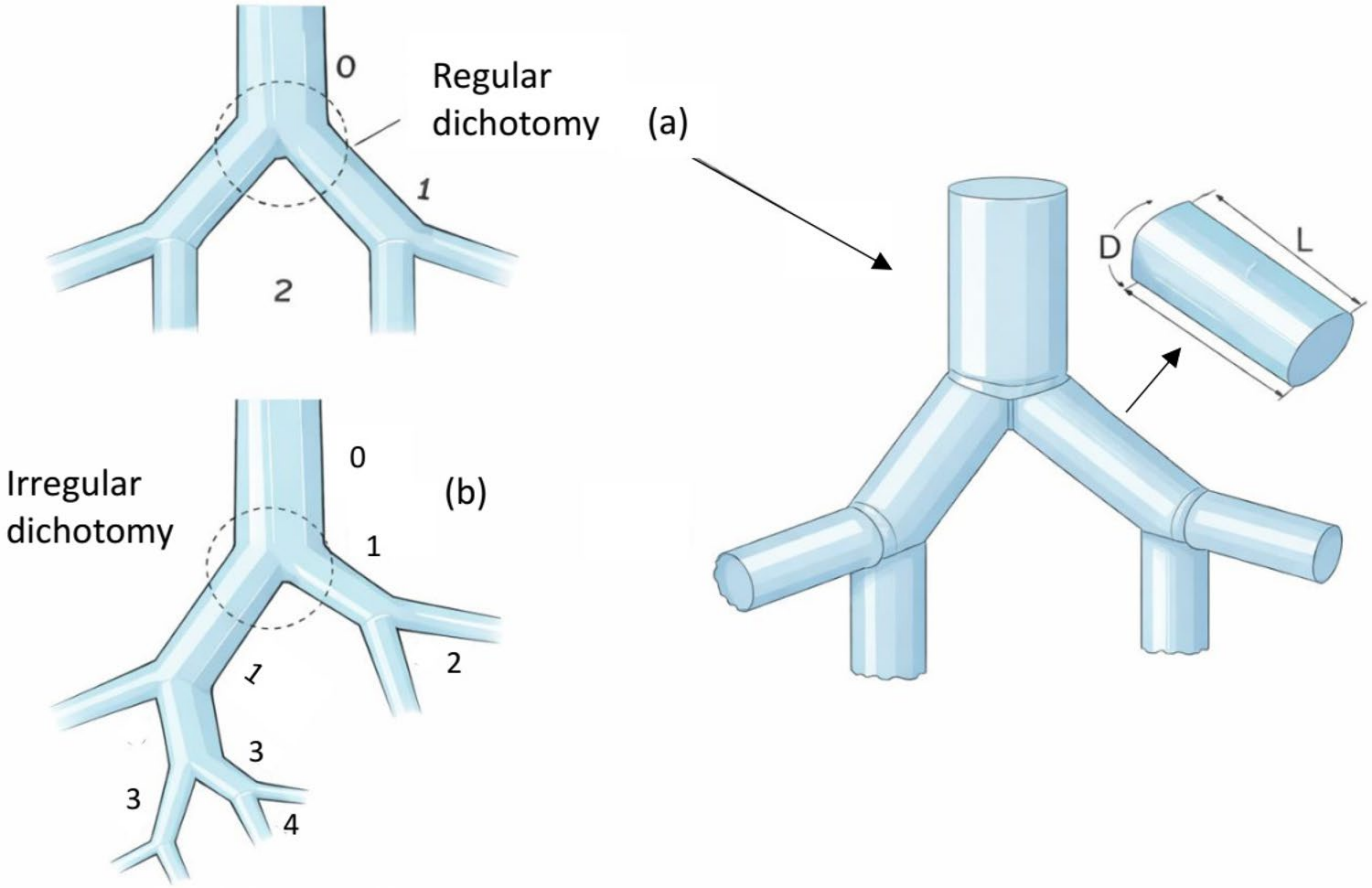
Weibel A model (**a**) Symmetric model (**b**) Asymmetric model (adapted from [16])

Early respiratory airway models primarily relied on simplified and symmetric representations to enable analytical tractability and reduced computational cost [18]. The classical Weibel model [16], based on cylindrical airways shown in Fig. 2 and idealized dichotomous branching, provided a convenient mathematical framework for studying global airflow distribution and average aerosol transport. However, compared with anatomically realistic geometries, such simplified models cannot resolve localized secondary flows, asymmetric branching effects, or heterogeneous deposition patterns that strongly influence regional drug delivery. Subsequent refinements, including the human bronchial tree proposed by Raabe in 1976 [19], improved morphometric realism while preserving computational efficiency, thereby establishing the foundation for many contemporary CFD studies.

Weibel’s Model A, shown in Fig. 2, reveals the lack of detailed information regarding the terminal bronchioles and alveoli in the modeling of airflow and particle behavior within the lung region. Thanks to progress in computed tomography (CT) and magnetic resonance imaging (MRI) technologies, 3D reconstruction of complex airway structures has now become largely feasible. Nevertheless, because CT imaging is more effective in resolving larger airways, this approach can accurately capture only the major segments of the bronchial branching architecture.

### ET Region

**Fig. 3:**
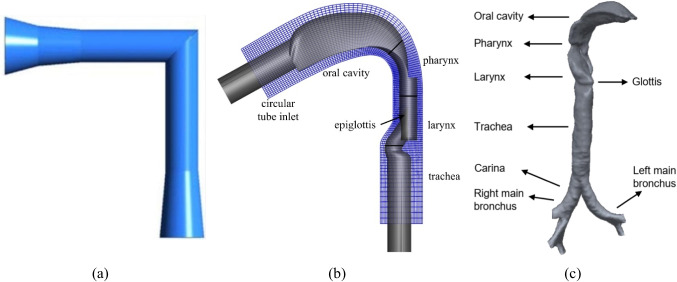
In *silico* models for the ET region: (**a**) USP induction port; (**b**) IMT model [20]; and (**c**) CT-based realistic MT airway model [21]

Airflow patterns in the extrathoracic region play a vital role in the transport of aerosols into the human respiratory system. Several studies have shown that, depending on the flow rate and particle’ size, the deposition in ET region can range from approximately 20% to 50% [3]. However, to prevent overdosing and to ensure effective therapy, deposition of medical aerosol particles in this region should be minimized. Figure 3 presents the idealized and realistic mouth-throat (MT) models used by the United States Pharmacopeia (USP) to represent the ET region.

The USP induction port shown in Fig. 3a was developed as a benchmark device in the pharmaceutical sector to evaluate the efficacy of inhalable drug formulations. This apparatus comprises a 90° curved section with a smooth circular profile and is widely used in research and development, particularly for numerical investigations of aerosol transport. However, as illustrated in Fig. 3a, the USP induction port does not incorporate key anatomical structures of the ET region including the oral cavity, epiglottis, oropharynx, and larynx. As a result, when *in vitro* and *in silico* findings are evaluated against *in vivo* measurements, the USP induction port inadequately represents aerosol deposition within the ET region. Consequently, this model has primarily been employed to assess drug delivery performance of inhaler devices rather than to replicate *in vivo* deposition characteristics.

The USP induction port has been widely adopted as a benchmark device due to its geometric simplicity and repeatability, making it particularly suitable for standardized *in vitro* testing and rapid numerical evaluations [22–25]. However, in contrast to anatomically realistic representations, the USP geometry omits critical structures such as the oral cavity, epiglottis, and laryngeal constrictions, which play a dominant role in jet formation and inertial impaction. Consequently, while the USP induction port provides consistent device-comparison metrics, it often underpredicts physiologically relevant deposition patterns. Alternative idealized mouth–throat designs therefore attempt to balance geometric simplification with the preservation of key anatomical features, enabling improved agreement with *in vivo* measurements while maintaining manageable computational cost [26].

In addition to the USP induction port and other idealized mouth–throat geometries, more anatomically representative *in vitro* models have been developed through collaborative efforts such as the Oropharyngeal Consortium (OPC). These models incorporate realistic oral cavity, pharyngeal, and laryngeal structures derived from imaging data and have been widely adopted within the pharmaceutical industry for standardized inhaler testing and regional deposition assessment. Compared with simplified USP-type inlets, OPC geometries provide improved agreement with *in vivo* and CFD predictions, thereby offering a more physiologically relevant platform for evaluating aerosol transport and drug delivery performance.

As shown in Fig. 3b, the idealized model was constructed at the Alberta Aerosol Research Laboratory using CT/MRI imaging data along with mean geometric parameters [20, 27]. In this model, the key anatomical characteristics are maintained, while geometrically complex regions that do not significantly influence airflow or particle deposition are simplified. The model has been utilized in studies on inhaler design as well as investigations of aerosol transport within the ET region under diverse conditions. In comparison to the USP induction port, it offers a more realistic representation of the ET region, thereby providing more reliable predictions of both regional and overall aerosol deposition.

Recent advances in CT and MRI imaging have enabled the construction of subject-specific, anatomically realistic ET geometries that substantially improve the fidelity of airflow and aerosol transport predictions. Several studies have demonstrated the benefits of such reconstructions. For example, Jayaraju *et al*. [28] developed MT models based on CT data from five healthy male subjects, where high-resolution imaging (0.3–0.5 mm) proved critical for accurately resolving airflow and deposition characteristics [29]. Additional investigations have reported realistic MRI-based models [30], COPD-specific geometries obtained using low-radiation scanning techniques [31], and pediatric oral airway replicas representing children aged 6–14 years [32]. Furthermore, Xi *et al*. [33] examined age-dependent variations in airflow and aerosol transport using multiple CT-based models corresponding to different age groups. Compared with idealized or standardized models, these realistic reconstructions capture complex curvature, asymmetry, and local constrictions that govern flow separation, turbulence generation, and regional deposition. As a result, they provide more reliable estimates of both regional and total deposition across diverse physiological and pathological conditions. However, this increased anatomical detail comes at the expense of higher computational cost and reduced generalizability, highlighting the ongoing trade-off between physiological realism and modeling efficiency.

Xi and Longest [34] derived three idealized geometries from their base model, i.e., circular, elliptical, and constant diameter. These geometries have been widely employed to investigate the influence of breathing profiles [35, 36] as well as the aerosol delivery efficiency of dry powder inhalers (DPIs) [37]. However, geometries derived from subject-specific data hinder the generalization of findings due to pronounced inter-individual anatomical variability. Consequently, the development and adoption of a standardized ET model remain necessary.

Tohidi *et al*. [38] demonstrated that nasal obstruction in an anatomically realistic nasal model significantly alters both airflow patterns and particle deposition. Their findings indicate that nasal obstruction is a determining factor in drug delivery efficacy and exposure to toxic particles. Liu *et al*. [39] investigated particle transport and deposition using a model based on pediatric ET geometry, providing fundamental insights for the optimization of pediatric inhalation therapies. Wu *et al*. [40] introduced an innovative approach to targeted aerosol drug delivery by demonstrating, within a realistic respiratory system model, that particles can be directed to a specific region of the left lung through magnetic targeting. As shown in Fig. 3c, Ciloglu [21] examined airflow characteristics and particle dynamics under steady and cyclic breathing conditions using a CT-based, anatomically realistic airway model.

### TB and Alveolar Region

In the tracheobronchial region, gas exchange is facilitated by the transport of airflow toward the alveoli. Determining aerosol transport and deposition within these critical regions is essential for improving drug delivery efficiency. Accordingly, CFD studies addressing this region have employed a range of approaches, from the basic Weibel model to anatomically realistic CT-based airway models.

**Fig. 4:**
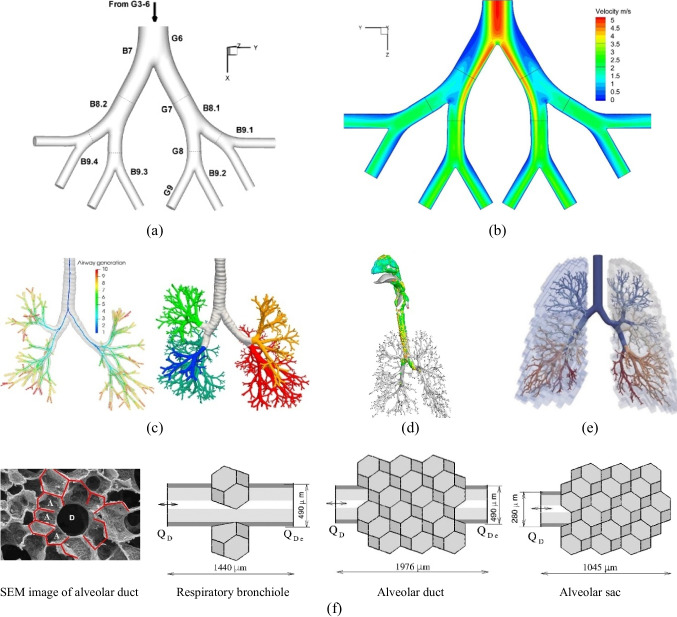
TB and alveolar models (**a**) TBU model [42], (**b**) velocity contours in an ideal G3-G6 TBU model [47], (**c**) CT-based TB airway tree and extended model [48], (**d**) particle deposition in a CT-based TB airway tree [49], (**e**) algorithm-based TB model [50], and (**f**) acinar region [51]

In this region, early modeling efforts largely relied on the classical Weibel Model A (Fig. 2), which represents the airway tree using symmetric, cylindrical, and dichotomously branching segments. This simplified framework enabled analytical formulations and computationally efficient simulations, making it particularly suitable for studying global airflow distribution and average deposition trends. However, compared with anatomically realistic geometries, such idealized representations cannot adequately capture asymmetric branching, localized flow separation, or inter-generational variability that strongly influence regional particle transport. Dimensional improvements by Finlay *et al*. have increased geometric consistency while maintaining computational applicability [41], thus both models and their geometric variants have been widely used in numerical and experimental research [42–44]. In this context, reduced-order representations such as triple bifurcation unit (TBU) models have emerged as practical compromises, allowing detailed investigation of local flow physics while maintaining manageable computational demands [45–47]. The computer-based models illustrated in Fig. 4 serve as fundamental tools for investigating airflow dynamics and aerosol behavior.

Numerous idealized airway geometries derived from the Weibel framework have been proposed to investigate specific transport mechanisms under controlled conditions. As shown in Fig. 4a, Kleinstreuer *et al*. [45] employed a TBU bronchial airway model to represent the TB region. Yu *et al*. [43] utilized a TB model spanning generations G0-G16 to examine droplet formation, as well as their transport and deposition during expiration. Koullapis *et al*. [48, 52] developed an idealized bronchial tree covering generations G10-G19 together with a heterogeneous acinar model. While these simplified configurations facilitate systematic parametric analyses and reduce computational cost, they inherently neglect anatomical irregularities and subject-specific variations that govern realistic deposition patterns. Consequently, such models are particularly valuable for isolating fundamental mechanisms, such as bifurcation-induced impaction or flow similarity across generations, but their predictive capability for patient-specific or regional dose estimation remains limited. This distinction underscores the complementary roles of idealized and realistic models in respiratory CFD research.

CT- and MRI-based anatomically realistic airway reconstructions have increasingly replaced idealized geometries for detailed investigation of airflow and aerosol transport in the tracheobronchial and distal regions (Figs. 4c-d). Islam *et al*. [53] investigated particle transport and deposition using an asymmetric airway model reconstructed from CT images and extending to generation G17. Their results indicated that the majority of particles are retained in the upper airways owing to inertial impaction, whereas smaller-diameter particles can be transported beyond G17 into distal regions, ultimately reaching the acinar zone. Similar anatomically realistic models have also been employed by Xi and Longest [34] and others, however, these studies have noted that pronounced inter-subject anatomical variability may limit the generalization of findings. More broadly, compared with symmetric or algorithmically generated trees, such subject-specific reconstructions preserve true airway asymmetry, curvature, and branching heterogeneity, leading to more accurate prediction of localized deposition and distal particle penetration. Nevertheless, the use of high-fidelity reconstructions introduces substantial computational cost and limits the feasibility of large parametric or population-based studies. As a result, a clear trade-off persists between anatomical realism and modeling flexibility, motivating the continued development of hybrid or reduced-order approaches that balance accuracy with efficiency.

Although numerical modeling increasingly favors airway geometries that are far more realistic than the Weibel model, capturing the distal generations containing millions of alveoli from CT images remains highly challenging. Due to their high level of anatomical detail, realistic models are not readily adaptable to parametric studies, which constitutes a significant limitation in investigations that require flexibility. Consequently, alternative models created through computational algorithms derived from CT imaging have been developed. These approaches enable anatomically more anatomically realistic representations compared to the Weibel model and allow for subject-specific simulations that can be adapted to disease conditions. For example, Monte Carlo-based 2D tree generation techniques proposed by Tawhai *et al*. [54] have been employed to construct 3D airway networks that closely resemble *in vivo* morphometry [55]. This method allowed the systematic evaluation of both normal lungs and lungs showing structural abnormalities such as asthma. Furthermore, as seen in Fig. 4e, temporally evolving 4D frameworks enable the integration of breathing motion and structural dynamics, providing a more realistic representation of airflow and deposition under cyclic conditions [50, 56]. Nevertheless, despite their efficiency and flexibility, these algorithmic models still rely on simplified assumptions [51] and may not fully capture fine-scale anatomical heterogeneity (Fig. 4f). Therefore, they are best regarded as complementary tools that bridge the gap between fully realistic imaging-based reconstructions and highly simplified analytical models.

### The Whole Lung Airway

Because the respiratory system comprises more than 16 million airways, its geometry is extremely complex, and with current technology it is not feasible to model the entire structure in full detail. Therefore, a variety of simplified airway models have been developed to preserve the essential anatomical and functional characteristics while maintaining reasonable computational costs. The whole-lung airway models (WLAMs) have been employed to investigate branching structures, airflow dynamics, and particle deposition. These models enable both simulations of overall lung function and detailed analyses of specific regions.

**Fig. 5:**
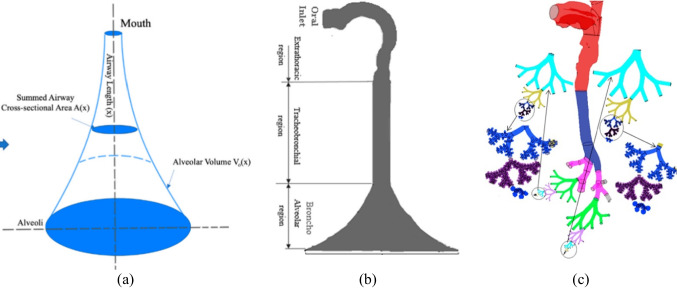
Lung models: (**a**) trumpet model [57]; (**b**) WLAM model [58]; and (**c**) 3D WLAM model with simplified TB and alveolar regions [59]

The trumpet model based on the Weibel geometry, shown in Fig. 5a, was developed by Yu [57] as a 1D system in which the cross-sectional area of each generation is conserved. The trumpet model describes how inhaled steady-state particles are transported and deposited in the airways using a mass balance method. Aerosol concentration is treated as a function of airway depth and time, and these variables are subsequently used to calculate regional deposition.

Kolanjiyil and Kleinstreuer [58] developed a simplified WLAM based on a realistic lung geometry, as illustrated in Fig. 5b. In this model, the region from the mouth to the trachea is represented in three dimensions, while the distal regions are simplified and, the MT region, where aerosol deposition is most pronounced, is modeled in a realistic manner. Realistic breathing scenarios can be generated through wall motion, and computational time is reduced by collapsing successive generations into a one-dimensional representation. However, the model does not adequately capture region-specific aerosol deposition or the detailed flow structures in the TB and alveolar regions, which are of particular importance for medical treatment. To achieve a more accurate representation of the respiratory system, the same authors subsequently developed an ET model spanning from the nasal and oral cavities to third generation, coupled with a model composed of TBUs spanning generations G4-G15 [59]. In addition, the model incorporates a double-bifurcation unit for generations G22-G23, as well as spherical alveoli that expand and contract during inspiration and expiration (Fig. 5c). This enhanced WLAM was designed to investigate particle transport and deposition throughout the breathing cycle while accounting for alveolar dynamics.

In addition to these multiscale and hybrid modeling strategies, airway morphology itself constitutes a major source of variability in predicted deposition outcomes. While parameters such as aerosol size distributions or breathing profiles can be adjusted relatively easily within conventional CFD frameworks, capturing intra- and inter-subject anatomical differences remains considerably more challenging. Even small variations in airway curvature, branching angles, or local constrictions may substantially modify flow separation, secondary motions, and particle trajectories, leading to pronounced changes in regional drug delivery efficiency. To address this limitation, recent studies have increasingly adopted patient-specific and morphology-aware modeling approaches based on imaging-derived geometries. Moreover, advanced techniques such as adjoint solver–based sensitivity analysis and shape optimization provide systematic and computationally efficient means to quantify how local geometric perturbations influence deposition metrics. Compared with conventional trial-and-error parametric studies, these gradient-based methods enable identification of critical airway regions and offer a more rational framework for geometry refinement and inhalation device design.

Building on these modeling advances, several research groups have made substantial contributions to the development and validation of whole-lung and multiscale respiratory CFD models. Notably, the group led by Ching-Long Lin has conducted extensive studies on image-based, subject-specific airway reconstructions and large-scale simulations of airflow and particle transport across the full respiratory tract [60, 61]. Similarly, the group of Worth Longest has produced numerous investigations integrating CFD with *in vitro* and *in vivo* measurements to evaluate inhaler performance, regional deposition, and therapeutic aerosol delivery [62, 63]. These studies have significantly advanced the translation of computational modeling toward realistic, clinically, and pharmaceutically relevant applications. Table 1 provides an overview of modeling approaches for airflow and aerosol transport in the human respiratory tract, highlighting their respective advantages and limitations.

**Table 1:** Advantages and limitations of respiratory airway modeling approaches reported in the literature

Model/Approach	Level of Representation	Advantages	Limitations	Typical Applications	References
Weibel A model	1D, symmetric TB-alveolar	• Simple and computationally efficient • Suitable for analytical formulations • Provides average regional deposition trends	• Lacks anatomical realism • Ignores airway asymmetry and upper airways • Cannot resolve local deposition	Theoretical analysis, preliminary assessments	Hofmann [15]; Weibel [16]
Trumpet model	1D, generation-based	• Mass balance-based formulation • Captures time-dependent transport	• Highly idealized geometry • No turbulence or local flow features	Average aerosol transport and deposition	Hofmann [15]; Yu [57]
Idealized models	3D, local	• Enables fundamental flow and deposition mechanism analysis • Well suited for parametric studies	• Limited physiological relevance • Results difficult to generalize to real lungs	Flow-particle interaction studies	Xi and Longest [34]; Kolanjiyil *et al*. [37]
USP/IMT models	3D, ET	• Standardized geometries • Suitable for experimental and CFD validation • Widely used in inhaler studies	• Simplified tongue and glottis representation • No subject-specific variability	Inhaler performance and deposition studies	Longest *et al*. [64]; Huang *et al*. [65]
CT-based realistic ET models	3D, ET	• High anatomical fidelity • Accurate prediction of turbulence and local deposition • Strong clinical relevance	• High computational cost • Significant inter-subject variability	Targeted drug delivery, patient-specific studies	Jayaraju *et al*. [28]; Nicolaou and Zaki [30]; Xi *et al*. [33]
CT-based TB models	3D, TB	• Realistic bifurcation geometry • Captures heterogeneous deposition patterns	• Limited distal airway coverage • Alveolar region typically excluded	TB-focused aerosol transport studies	Kim *et al*. [66]; Poorbahrami and Oakes [67]
WLAMs	Hybrid (3D/1D)	• Represents the entire lung • Enables lobar and regional dose estimation	• Loss of local flow and deposition details • Simplified alveolar representation	Exposure and dosimetry analysis	Hofmann [15]; Kolanjiyil and Kleinstreuer [58]
Advanced WLAMs (Dynamic alveolar models)	Hybrid, transient	• Incorporates breathing cycle effects • Improved physiological realism	• Increased model complexity • Higher computational demand	Cyclic breathing and transient deposition	Kolanjiyil and Kleinstreuter [59]
Monte Carlo/Algorithmic lung models	Stochastic/generative	• Captures airway asymmetry and variability • Adaptable to diseased lungs	• CFD coupling is challenging • Limited experimental validation	Virtual population and sensitivity studies	Kitaoka et al. [50]; Tawhai *et al*. [54]; Martonen *et al*. [56]
Acinar honeycomb/polyhedral models	3D, alveolar	• Resolves intra-acinar flow structures • Accurate sedimentation and diffusion prediction	• Simplified alveolar morphology • Not representative of the full lung	Micro-scale aerosol transport	Kumar *et al*. [51]; Ciloglu [68]

## Modeling of Airflow and Particle Dynamics Using CFD

Accurate prediction of airflow and aerosol particle transport within the respiratory airways depends not only on the numerical method employed but also critically on the suitability of selected turbulence model. In ET region of the human respiratory system, complex flow phenomena such as high velocity gradients, jet formation, and flow separation are commonly observed. In contrast, the TB and alveolar regions are characterized by low Reynolds number flows that are viscosity-dominated and predominantly laminar in nature, exhibiting markedly different flow dynamics [44]. Consequently, applying a single turbulence modeling approach uniformly across the entire respiratory tract is not physically appropriate and may compromise predictive accuracy.

### Fundamental Characteristics of Airflow in the Respiratory Airways

In upper respiratory region, especially downstream of the glottal constriction, airflow reaches high velocities and exhibits a jet-like behavior. This jet flow leads to flow separation, recirculation, and the formation of unsteady vortical structures over relatively short distances. In this region, Reynolds numbers typically correspond to the turbulent flow regime. In contrast, as airway diameters decrease and flow velocities diminish in the TB and alveolar regions, the Reynolds number decreases markedly, and the airflow is predominantly laminar or within the transitional regime. Moreover, the inspiration-expiration cycle induces time-dependent flow structures, especially in the distal regions of the respiratory tract.

Most respiratory CFD studies assume rigid airway walls for computational simplicity. However, the human respiratory system is inherently dynamic, with cyclic expansion and contraction of the airways and alveolar regions during breathing. To better represent physiological conditions, several investigations have incorporated moving-wall formulations, deforming meshes, or fluid–structure interaction (FSI) techniques to account for airway compliance and tissue mechanics [69–71]. Compared with rigid-wall assumptions, these approaches enable more realistic prediction of transient flow structures, regional ventilation, and particle deposition patterns, particularly in the distal lung where wall motion significantly influences transport behavior. Although such models remain computationally demanding, they provide an important step toward physiologically faithful simulation of inhalation drug delivery and exposure assessment.

### Computational Approaches for Turbulent Flow Simulation

Turbulent respiratory airflow has been modeled using three principal CFD strategies: direct numerical simulation (DNS), Reynolds-averaged Navier–Stokes (RANS), and large eddy simulation (LES). Although DNS offers the highest fidelity, its computational requirements render it impractical for realistic, multiscale airway geometries [12, 30]. Consequently, most respiratory simulations rely on RANS or LES formulations, which provide more feasible compromises between accuracy and cost.

RANS-based models remain attractive due to their low computational demand [25]; however, their time-averaged formulation often limits their ability to resolve transient vortical structures, post-glottal jet breakdown, and flow separation in the extrathoracic region [23, 27, 41, 72, 73]. Among these models, k-ω and SST variants generally outperform the conventional k-ε formulation in near-wall and low-Reynolds-number conditions and have demonstrated improved agreement with deposition experiments in realistic airway geometries [8, 28, 52, 58, 59, 74–76]. Nevertheless, all RANS formulations may underpredict highly unsteady flow features.

In contrast, LES directly resolves large-scale turbulent structures and has consistently shown superior performance in capturing jet instabilities, secondary flows, and particle–flow interactions in the upper airways [6, 22, 31, 53, 77–79]. Several studies report closer agreement with experimental deposition measurements compared with RANS approaches [80, 81]. However, the substantially higher computational cost of LES restricts its routine application to localized or high-fidelity investigations.

As a result, these findings indicate that no single turbulence model is universally applicable across the entire respiratory tract [44]. Instead, model selection should be guided by local Reynolds number, geometric complexity, and the desired balance between predictive accuracy and computational efficiency.

### Decision Analysis for CFD Modeling

In CFD-based studies of airflow and aerosol transport in the respiratory system, turbulence model selection should not follow a one-size-fits-all approach but instead be guided by the flow characteristics of the region under investigation. In the upper respiratory tract, particularly in configurations dominated by post-glottal jet formation, flow separation, and strong velocity gradients, the LES approach should be preferred to accurately resolve unsteady vortex structures. By contrast, in cases where the upper airway geometry is relatively smooth and pronounced jetting or separation is absent, *k-ω* or LRN RANS models can provide acceptable levels of accuracy.

In the TB and alveolar regions, airflow generally exhibits LRN, viscosity-dominated behavior. Accordingly, a laminar flow assumption is often sufficient at low Reynolds numbers, while *k-ω*-based RANS models offer an appropriate balance between computational cost and accuracy at higher Reynolds numbers. Furthermore, in the studies examining the time-dependent effects of the respiratory cycle, a transient solution strategy should be adopted regardless of the selected turbulence model. Overall, turbulence model selection should be treated as a region-specific and physics-based decision process along the respiratory tract.

### Modeling of Aerosol Dynamics

**Fig. 6:**
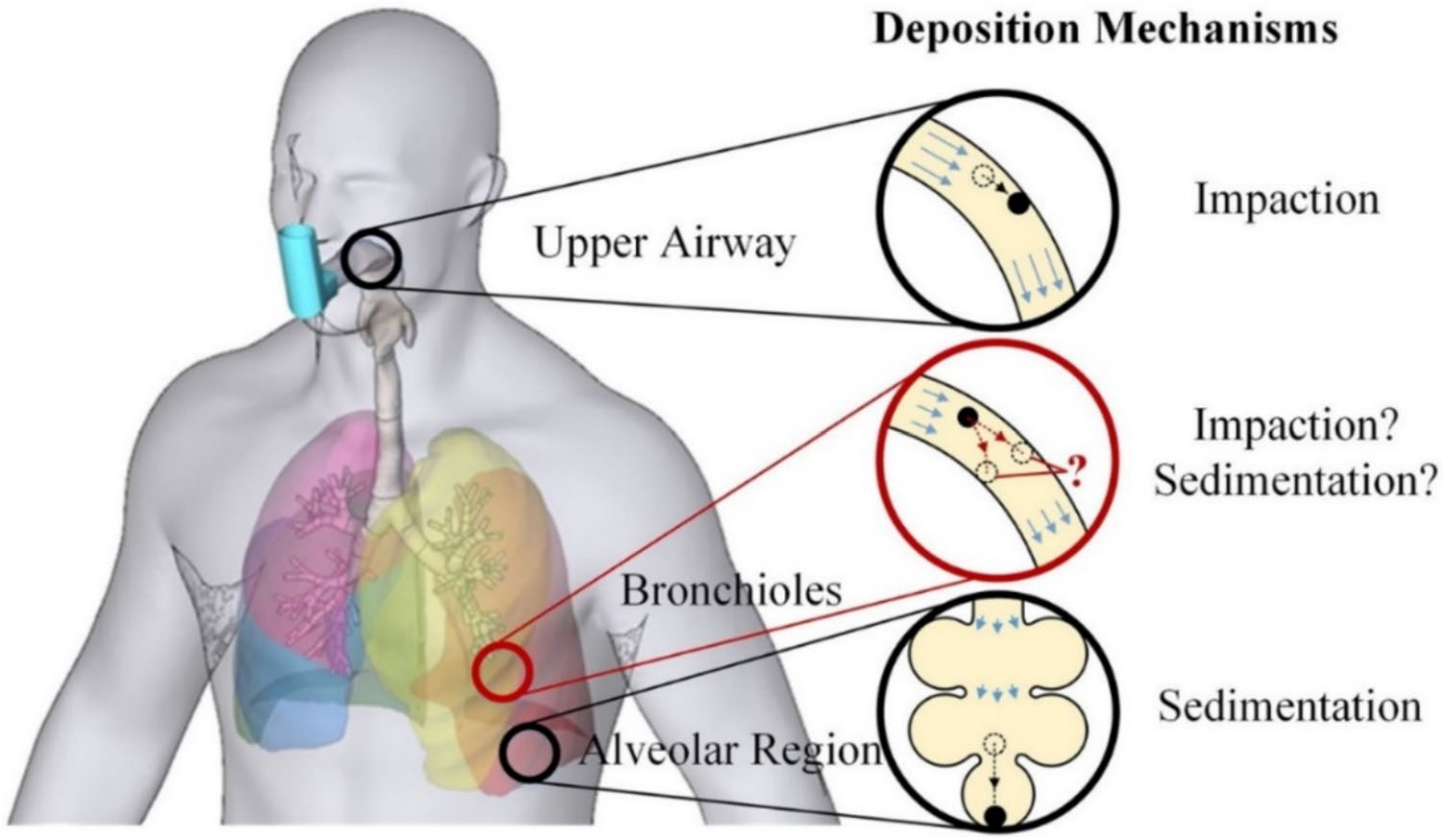
Schematic representation of the particle deposition mechanisms in the human respiratory tract [83]

Particles deposit in different regions of the respiratory system through various mechanisms depending on their aerodynamic properties. The main mechanisms of particle deposition include inertial impaction, sedimentation, diffusion, interception, and electrostatic precipitation [15]. In particular, larger particles are unable to follow rapidly changing airflow paths due to their inertia at high flow velocities, leading to collisions with airway walls in curved passages; this mechanism is predominantly active in the upper respiratory tract (Fig. 6). The influence of gravity on particle deposition depends on both particle size and residence time within the airways. Conditions such as breath holding increase particle residence time in the lungs, thereby enhancing sedimentation. Studies conducted by NASA have further reported that particle behavior is significantly altered in low-gravity environments, such as space, highlighting the critical role of gravitational effects in aerosol dynamics [82]. Table 2 presents the dominant aerosol deposition mechanisms as a function of particle size and airway region.

**Table 2:** Relationship between aerosol size and deposition mechanisms

Reference	Particle Size	Dominant Mechanism	Deposition Region	Behavior
Taulbee and Yu [84]	< 0.1 µm	Diffusion	Alveolar	Deep penetration
Zhang *et al*. [85]	0.3 µm	Minimum deposition	TB-ET	Critical size
Robinson *et al*. [86]	1–5 µm	Sedimentation	TB	Moderate deposition
Ciloglu [87]	> 5 µm	Impaction	ET	High deposition

Brownian diffusion, which dominates for particles smaller than approximately 0.5 µm, arises from random collisions between particles and airway walls. Fibrous or elongated particles may also deposit through direct contact with airway surfaces while following the airflow, owing to their geometric characteristics. This mechanism is referred to as interception and is more effective for larger or elongated particles. Particles with diameters greater than 10 µm primarily settle in the upper respiratory airways, whereas particles in the 5–10 µm range mainly deposit in the lower airways, and particles between 0.5 and 5 µm tend to reach and deposit in the alveolar region. In addition, electrically charged particles may be attracted to or repelled from airway surfaces due to electrostatic forces. Consequently, reducing the electrical charge of aerosols prior to inhalation has been recommended as an effective strategy for controlling particle deposition [88].

In modeling aerosol particles within the human respiratory system, either Euler-Euler or Euler–Lagrange approaches are commonly employed. In the Eulerian framework, aerosols are treated as a separate continuous phase, and their behavior is governed by the solution of mass and momentum conservation equations. However, this approach becomes inadequate in situations where strong gas-aerosol interactions occur or where accurate representation of discrete phase removal is required, as individual particle motions and interactions can only be captured in a limited manner. Consequently, the Eulerian approach does not always provide sufficient accuracy for aerosol-phase predictions.

In contrast, the Lagrangian approach treats the aerosol phase as a collection of discrete particles embedded within a continuous gas phase, offering a more natural and detailed description of particle dynamics. The trajectory of each particle is solved based on Newton’s second law of motion [24], enabling more precise predictions of aerosol transport and deposition. Although tracking many particles increases computational cost, the Lagrangian approach yields physically more realistic and interpretable results. The Euler–Lagrange approach, which enables a detailed representation of interphase interactions, can be broadly classified into two main categories: the Discrete Phase Model (DPM) and the Discrete Element Method (DEM).

#### DPM Approach

In many studies employing the DPM, the system is considered one-way coupled, assuming that the dispersed phase is dilute and does not affect the carrier flow; consequently, particle–particle interactions are neglected [24]. In addition, particle deposition is typically assumed to occur upon wall contact. In the ET region, the primary mechanisms for micron-sized particle deposition are inertial impaction and sedimentation due to gravity. While impaction dominates in high-velocity regions, gravitational effects become more pronounced for larger particles. Accordingly, the contributions of pressure-gradient, added-mass, and Basset forces on micron-sized particles are generally considered negligible and are therefore omitted in most studies.

While many respiratory aerosol simulations adopt a one-way coupling assumption, in which particles are transported by the carrier airflow without affecting the flow field, this simplification may become invalid at high particle loadings. Under dense aerosol or powder delivery conditions, momentum exchange between the dispersed and continuous phases can significantly modify local velocity profiles and turbulence structures. In such cases, two-way coupling formulations provide a more physically consistent framework by accounting for the mutual interaction between particles and the airflow, thereby improving predictions of deposition and transport behavior [46].

In the literature, particle motion is most modeled using drag and/or gravitational forces [10, 22, 28, 35, 52, 58, 59, 80, 89]. Some studies have considered only gravitational forces [25, 65], whereas others have accounted solely for drag forces [53]. A limited number of studies have included the Saffman lift force; however, due to the much higher density of particles compared to air, its influence in the ET region has generally been reported to be weak [52]. In contrast, Kolanjiyil and Kleinstreur [58] reported that radial forces may enhance particle deposition, particularly along curved surfaces.

Zhang *et al*. [11] compared Breezhaler® and Handihaler® inhalers using a CFD-DPM framework under varying pressure drops and particle sizes. Their results indicated that Breezhaler® provides a more homogeneous deposition pattern, particularly in the TB region, whereas Handihaler® exhibits higher deposition in the oral cavity and deep lung. Accordingly, Handihaler® was found to be more effective for deep lung targeting, while Breezhaler® showed improved performance in the upper and central airways for smaller particles. Jiang *et al*. [90] examined the deposition behavior of micron-scale lunar dust particles in a full-lung model using numerical simulations combined with machine learning techniques, demonstrating that particles predominantly deposit in the upper and peripheral airways, with deposition patterns strongly dependent on breathing conditions and particle size. In another CFD-DPM study, Aboelezz *et al*. [5] validated numerical predictions of particle deposition in bronchioles against experimental data and showed that realistic, complex bronchiolar geometries provide greater interaction surface area than simplified models, resulting in increased particle retention.

#### DEM Approach

In CFD-DPM simulations, particle/particle interactions are generally neglected; however, at high particle concentrations, particle/particle, particle/fluid, and particle/wall interactions must be explicitly considered. Such conditions can be modeled through the coupled use of CFD and the DEM. For dry powder inhalers, particle–particle and particle–wall interactions play a critical role in determining powder dispersion, deagglomeration, and drug delivery efficiency. To address these mechanisms, DEM has increasingly been employed to explicitly resolve inter-particle collisions, agglomeration, and carrier-drug detachment processes. Compared with point-particle approximations, DEM provides a more realistic representation of granular dynamics and offers valuable insights into inhaler design and formulation optimization [4]. In DEM approach, the particles are treated as discrete entities, and their motion is computed based on Newton’s second law. In addition to mechanical contact forces, interparticle forces such as van der Waals, capillary, and electrostatic interactions are also considered.

Tong *et al*. [91] demonstrated that CFD-DEM coupling provides a more realistic representation of fluid-particle interactions. In these models, flow field information obtained from CFD is transferred to the DEM solver, and the two-way coupling is updated dynamically over time. Although CFD-DEM approach has found widespread application in the development of inhalable drug delivery systems, its use in modeling aerosol transport within the respiratory airways remains limited. Islam *et al*. [92] reported that CFD-DEM predicts more complex particle trajectories than CFD-DPM, particularly in the supratracheal region. Similarly, Feng and Kleinstreuer [46] and Kim *et al*. [93] demonstrated that CFD-DEM is effective in evaluating particle deposition and inhalation device performance in the ET and TB regions.

In summary, while the DPM approach assumes a dilute particle phase and neglects particle–particle interactions, the DEM approach explicitly resolves these interactions. Consequently, DPM is well suited for flows with low particle volume fractions, whereas DEM is preferred for high particle concentrations and problems where contact mechanics play a dominant role. However, despite its higher accuracy in interaction-dominated regimes, DEM requires substantially greater computational resources compared to DPM.

## Effect of Airway Geometry

The geometric characteristics of the respiratory airways constitute fundamental parameters that govern airflow structure and, consequently, the transport and deposition behavior of aerosol particles. Geometric features such as airway diameter, curvature, degree of constriction, and bifurcation angles directly influence whether the flow remains laminar or becomes turbulent, as well as local velocity gradients and vortex formation.

### Effect of the ET Region

The extrathoracic region of the upper respiratory system is an anatomical domain in which a substantial fraction of inhaled particles is retained and where the airflow field exhibits the most complex characteristics. In their study, Allen *et al*. [94] contrasted the upper airway geometry of a five-year-old child with an adult model, demonstrating that although the laryngeal jet axial velocities were comparable, the adult model exhibited higher Reynolds numbers and turbulence intensities. The findings highlight that ET region geometry plays a critical role in shaping flow patterns and turbulence characteristics. Xi and Longest [34] investigated the effect of realistic modeling of the MT region on aerosol deposition and reported that particle deposition was 25–40% higher in realistic geometries than in the USP induction port [64]. Similarly, Huang *et al*. [65] compared USP-, IMT-, and BT-based idealized models with realistic MT geometries and reported that realistic MT models exhibited smoother flow fields and a more uniform spatial distribution of aerosol deposition, while the total deposition amounts were comparable to those obtained with idealized models.

**Fig. 7:**
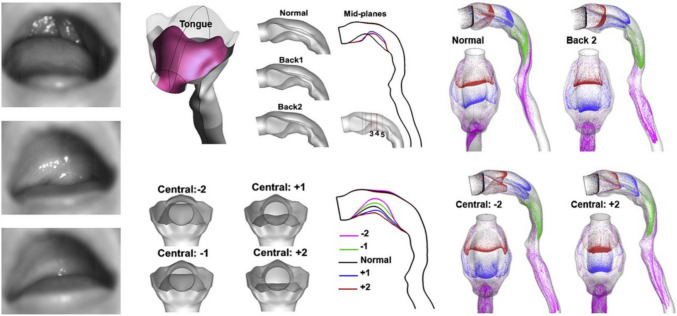
Vortex structures in the oropharyngeal airway at different flow rates for tongue positions. Red areas indicate a flow rate of 60 L/min, and blue areas indicate a flow rate of 15 L/min [96]

The influence of local geometric parameters within the ET region has also been examined in detail. Xi *et al*. [95], through CFD analyses conducted on realistic MT models incorporating different glottal variations, demonstrated that glottal area and airway volume are the primary geometric factors influencing aerosol deposition. In particular, the glottal constriction was shown to significantly increase aerosol deposition due to elevated flow velocities and enhanced impaction mechanisms. These results indicate that the ET region acts as a primary filter for aerosol transport and that geometric simplifications in this region may introduce substantial uncertainties in deposition predictions. Nicolaou and Zaki [30] further demonstrated that MT geometries from different individuals exert pronounced effects on velocity profiles and turbulence intensity, emphasizing the critical role of anatomical variability in aerosol transport and deposition. Xi and Yang [96] showed that tongue position markedly affects the airflow and particle dynamics within ET region, with oral deposition being dominant (Fig. 7).

The dimensions and morphology of ET region vary with age. Carrigy* et al*. [3] reported that ET deposition in children is more sensitive to flow rate than in adults and that a ten-year-old child exhibits higher ET deposition. They also noted that it is difficult to characterize the effects of ET geometric variations on aerosol dynamics using a single correlation. Ruzycki *et al*. [97] analyzed the uncertainties in the correlation proposed by Golshahi *et al*. [29] and developed a method that more accurately predicts ET deposition of nebulized aerosols in adults. The applicability of this approach to other aerosol types and pediatric patient groups may be explored in future* in silico* studies.

Using high-resolution LES-based CFD models, Sadeghi *et al*. [6] investigated aerosol drug deposition in a pediatric MT geometry and developed a logarithmic relationship between the Stokes number and drug deposition in the oral region. In their study, the effect of tongue position was systematically evaluated for the first time, revealing that positioning the tongue 4 mm lower increased pulmonary drug delivery by 4.6% under an asthmatic breathing profile.

### Effect of the TB and Alveolar Regions

Hofmann [15] investigated the particle deposition using different WLAM models. These models were shown to provide valuable insights into individual airways and generations depending on the size of the particles and the respiratory pattern. However, a fundamental limitation of whole-lung models is that their predictions can be validated against *in vivo* data only at the total and regional deposition levels, with no possibility for validation at the level of individual airways.

The influence of TB and combined airway geometries on aerosol deposition has also been emphasized. Feng *et al*. [98] demonstrated that upper airway morphology plays a decisive role in aerosol transport and deposition, reporting glottal constriction as the most dominant factor affecting particle deposition. Geometric variations in the upper airways were shown to alter velocity distributions, flow direction, and turbulence structures in the TB airways, thereby influencing aerosol transport. Similarly, Rahimi-Gorji *et al*. [99] reported that maximum velocity variations occur in constricted throat regions.

Kim *et al*. [66] compared two Weibel-based, computer-generated airway models and demonstrated that TB morphology strongly affects aerosol distribution, with higher deposition observed in the bifurcating regions of the lower airways. These findings were further supported by Poorbahrami and Oakes [67], who examined lung models from three different female subjects and reported that inter-subject geometric differences play a determining role in the particle deposition. In comparison with the idealized Weibel model, realistic geometries produced more complex flow patterns and more heterogeneous deposition distributions.

Dong *et al*. [100] compared a parametric Kitaoka model with a CT-based realistic TB model and showed that Brownian diffusion is the dominant deposition mechanism for ultrafine particles smaller than 10 nm, whereas deposition efficiency reduces in the range size of 10–100 nm. Christou *et al*. [101] analyzed sex-dependent airway morphometric differences using CT data from 185 individuals, reporting larger lumen areas in the upper airways of males and shorter bronchial segments in females. These findings highlight the importance of sex as a factor in respiratory dynamics and inhalation therapies.

Islam *et al*. [102] investigated particle deposition in lung models representing different age groups and demonstrated that deposition efficiency increases with both age and flow rate. The complex flow structures were observed in the MT and tracheal regions at high inhalation velocities, especially. These results provide insight into the influence of age-associated lung alterations on aerosol drug delivery.

**Fig. 8:**
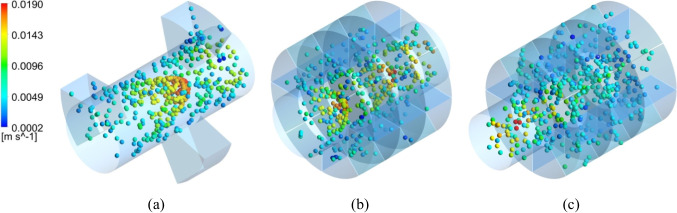
Aerosol deposition in (**a**) G17, (**b**) G18 and (**c**) G23 alveolar models for a flow rate of 15 L/min [104]

Haber *et al.* [103] examined airflow and aerosol deposition in a rhythmically expanding and contracting hemispherical alveolus model under low Reynolds number and quasi-steady flow assumptions. The study elucidated how particle transport and deposition mechanisms at the alveolar level evolve under time-dependent flow and geometric conditions. In a study employing idealized models with moving walls to represent the alveolar region (Fig. 8), it was reported that alveolar wall motion facilitates the delivery of particles to the distal regions of the lung [104].

## Effect of Airflow

### Airflow Rate

The flow rate is a key parameter controlling dust and aerosol deposition within the respiratory system. As the flow rate increases, turbulence intensity and particle inertia rise, which in turn enhances aerosol deposition, particularly in ET region. According to Kleinstreuer and Zhang [36], an increase in flow rate promotes turbulence formation based on laryngeal geometry, which in turn influences particle dynamics. Similarly, Jayaraju* et al*. [28] reported that total particle deposition increases with flow rate in a CT-based ET airway model, with deposition predominantly concentrated in the oral region. Longest *et al*. [64] employed TBU models corresponding to generations G3-G5 and G7-G9 to investigate the deposition of 1–7 µm particles during exhalation, solving the Navier–Stokes equations using CFD under laminar flow assumptions. Their results showed that deposition in the TBU region depends on the Dean number, while increasing Stokes number leads to a reduction in deposition efficiency.

Koullapis *et al*. [52] demonstrated that local deposition differences downstream of the main bifurcation originate from filtering effects in the MT region of the upper airways. At low inhalation flow rates, the prolonged residence time and enhanced Brownian motion increase the deposition of small particles, whereas the deposition of larger particles in MT region reduces [35]. Shang *et al*. [105] found that under two inhalation flow rates, notable secondary flows occur mainly in the larynx–trachea segment and the left main bronchus in a WLAM model. While these secondary flows significantly affect particle transport and deposition, more uniform flow patterns were observed in the terminal airways of the right lower lobe.

**Fig. 9:**
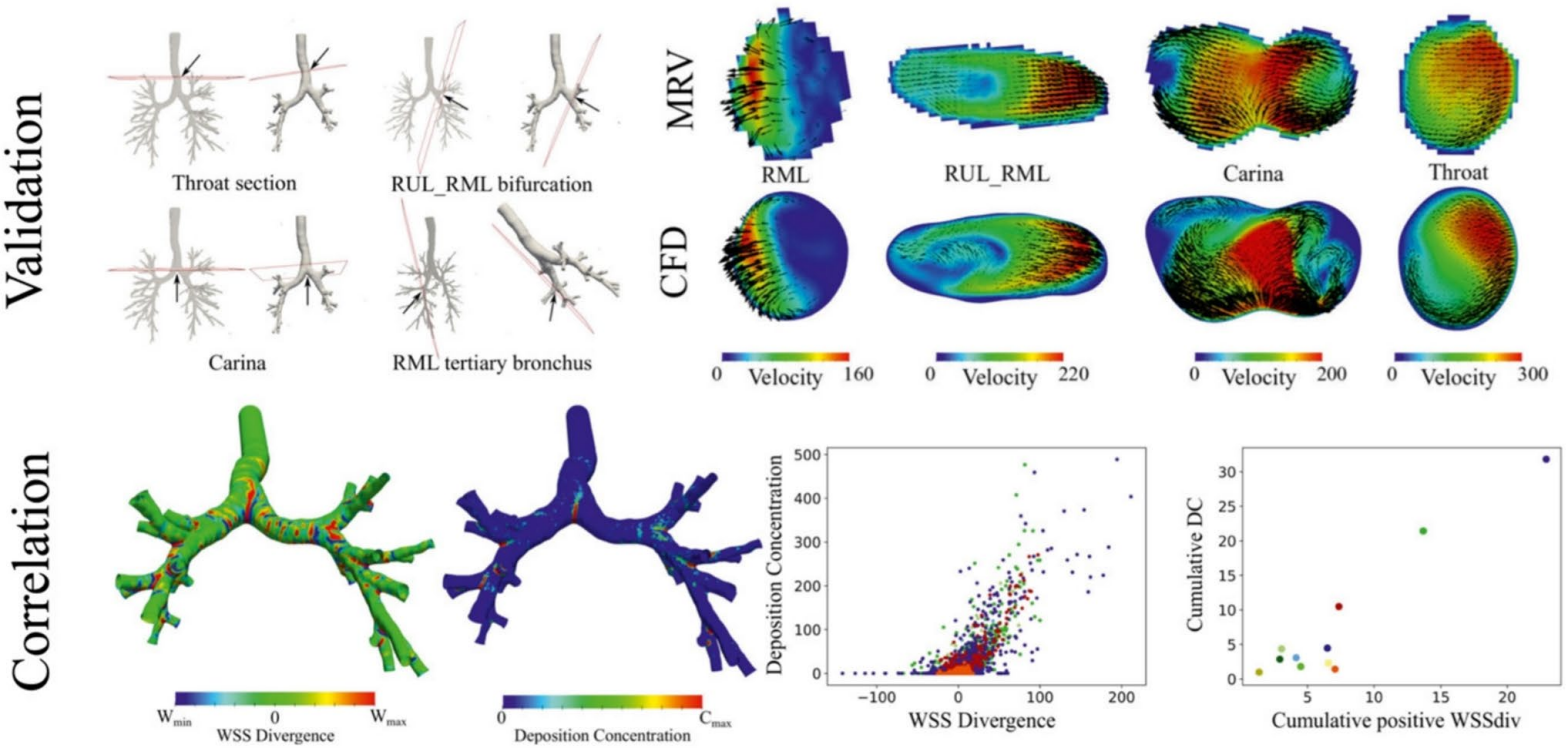
Velocity magnitudes and secondary flow motion patterns for CFD and MRV predictions [79]

**Fig. 10:**
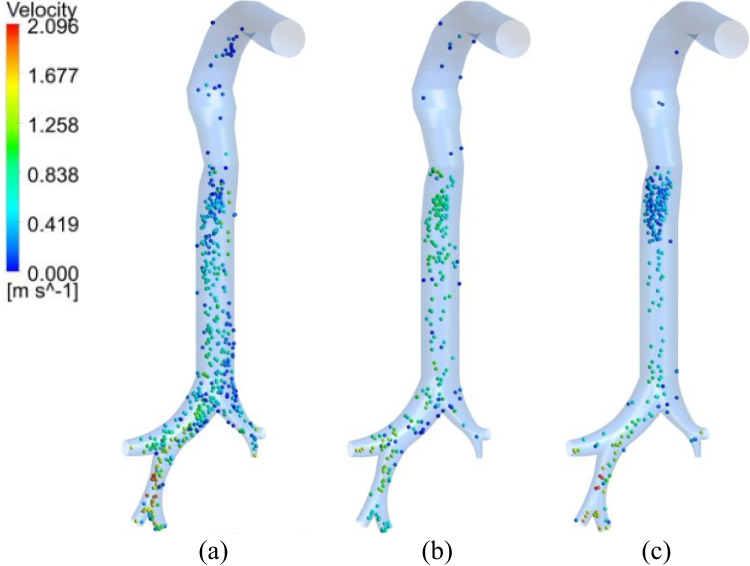
Distribution of 7 μm diameter aerosols at different flow rates (**a**) 15 L/min, (**b**) 30 L/min and 60 L/min [87]

On the other hand, airflow rate also influences laryngeal jet behavior. Cui *et al*. [81] demonstrated that under light breathing conditions (15 L/min), a longer and more stable jet reduces wall impingement, whereas under heavy breathing (60 L/min), the jet becomes unstable and interacts with recirculatory structures in the trachea, thereby increasing particle residence time and deposition. Farghadan *et al*. [79] proposed the wall shear stress divergence (WSSdiv) parameter to predict particle deposition in TB airways. CFD and LES analyses revealed strong correlations (Pearson > 0.7–0.9) between normalized deposition and positive WSSdiv values (Fig. 9), indicating that WSSdiv can be used to estimate surface deposition and regional dosimetry without explicitly resolving particle transport. Using a CT-based realistic airway model (Fig. 10), Ciloglu and Karaman [87] reported that increasing flow rate reduces the number of particles retained in the system, and that deposition efficiency decreases with increasing particle size. They further emphasized that deposition is higher during the inhalation phase, that inertial effects become more pronounced at high flow rates, and that the interplay between flow dynamics and particle properties plays a critical role in aerosol drug delivery.

### Transient Flow

Steady and time-dependent flow rates represent the two most investigated breathing patterns in particle deposition studies. Grgic *et al*. [106] compared these two flow types and demonstrated that unsteady flow leads to higher particle deposition, particularly in the MT region and, with increasing flow rate, in the vicinity of the larynx. Cui and Gutheil [78], through LES studies in an MT model, showed that vortex formation occurs under both flow conditions, while vortical structures are more complex in unsteady flow. With increasing inspiratory flow rate, the number of vortices increases while their characteristic lengths decrease, resulting in enhanced aerosol deposition. Under cyclic breathing conditions, the flow accelerates and transitions to turbulence, and during the deceleration phase it relaminarizes, with turbulence being more pronounced during this latter phase. Using idealized acinar region models, Ciloglu [104] reported that under time-dependent breathing conditions, particle deposition increases with flow rate, with total deposition efficiencies of 24%, 47%, and 77% at 15, 30, and 60 L/min, respectively.

### Breathing Pattern

Breathing patterns, particularly inhalation duration, exert a decisive influence on particle transport and deposition within the respiratory system. Based on Weibel’s symmetric lung model, Choi and Kim [107] developed a dynamic single-path mathematical model capable of analyzing particle deposition in the lungs under different breathing conditions. The model was shown to provide a more effective and detailed assessment of particle deposition compared with previous approaches. Park *et al*. [108] demonstrated that particle concentration retained in the deep lung regions increases with successive breathing cycles, but reaches saturation after the eighth breath. In contrast, Zhang and Kleinstreuer [109] reported that successive breathing has a limited effect on nanoparticle deposition. The authors noted that turbulent kinetic energy increases rapidly during the acceleration phase due to a rise in Reynolds number, while turbulence persists for a longer duration during the deceleration phase. Using simulations in three different realistic airway models, Lizal e*t al*. [110] examined the effects of airway generation number and flow velocity on particle deposition and compared the results with those obtained from the Weibel A model. Their findings indicated that airway geometry and flow conditions play a dominant role in particle transport.

Longest *et al*. [63] compared predicted deposition for DPIs in an adult whole-airway model with *in vivo* data and evaluated the effects of different inhalation waveforms. They showed that the breath-holding technique recommended for certain DPI devices significantly affects particle deposition. Using an airway model extending from the mouth to the G7, Kadota *et al*. [111] demonstrated that breath holding enhances turbulence and vortex formation, thereby reducing oral deposition while increasing particle deposition in the bronchi. This behavior was suggested to facilitate deeper penetration of particles into the airways, potentially enhancing the therapeutic efficacy of DPIs. Calmet *et al*. [112] investigated the behavior of inhaler aerosols in the nasal region under normal and exercise breathing conditions. Their results showed a marked increase in turbulence and particle deposition during exercise, with total deposition increasing fivefold and sinus deposition increasing sevenfold. These findings indicate that nasal drug delivery may have particularly high therapeutic potential during exercise.

**Fig. 11:**
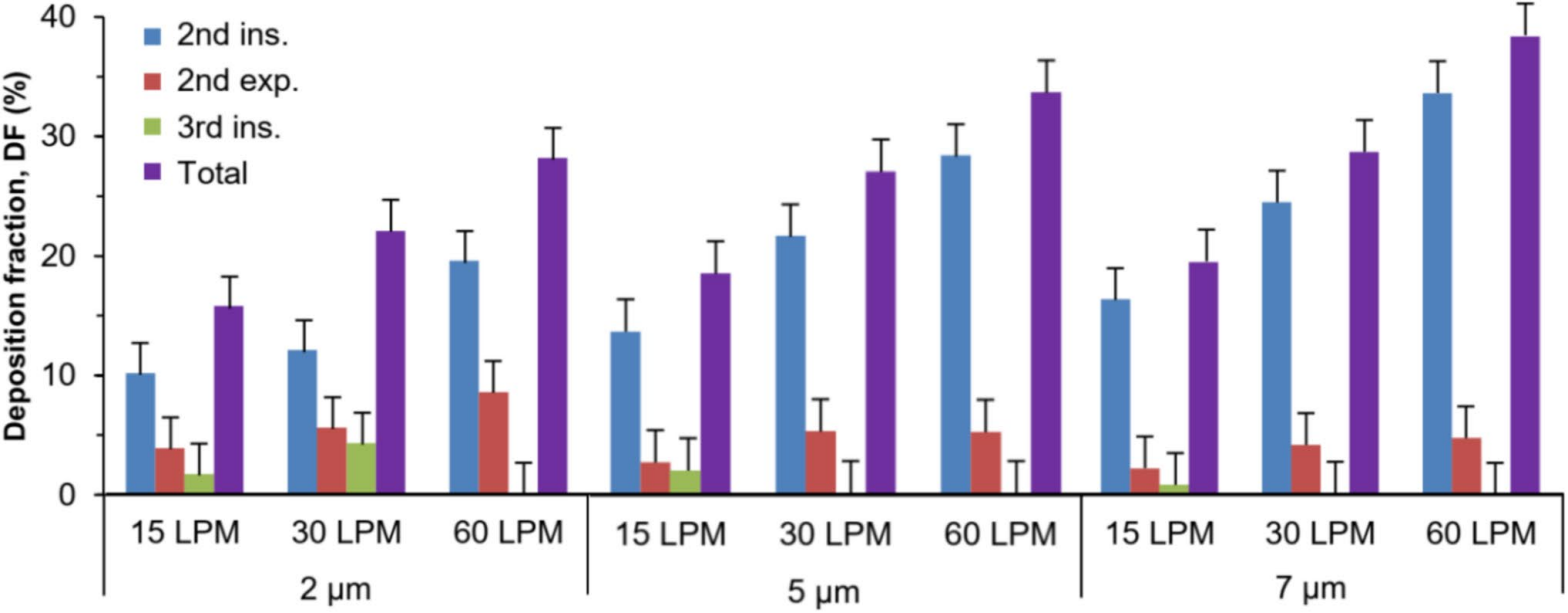
Deposition rates of aerosol particles (d_p_ = 2, 5 and 7 µm) during successive respiration cycles [87]

In CFD analyses conducted by Ciloglu [68] on a respiratory bronchiole model in the acinar region, vortical and asymmetric flow structures were observed within the alveoli, and aerosol deposition was reported to decrease with increasing particle size and flow rate. Larger particles tended to deposit along the channel walls, whereas smaller particles accumulated within the alveolar cavities. It was also emphasized that particle residence time increases during exhalation due to gravitational effects. In a subsequent study, Ciloglu and Karaman [87] showed that particle deposition during repeated breathing cycles increases with inspiratory flow rate. Due to high inertia, particles of 7 µm size and larger were found to deposit predominantly in the upper airway generations, and under heavy respiratory conditions, deposition efficiencies of 28.5%, 33.05%, and 38.4% were reported for 2, 5, and 7 µm particles, respectively (Fig. 11).

Mirzaaghaian *et al*. [113] analyzed the transport and deposition of various pollutants in the lungs under realistic breathing profiles representing different activity levels. Their findings showed that particle size and type are key determinants of deposition, particularly in the ET region. Moreover, larger and traffic-related particles exhibited increased deposition at high activity levels, and both the magnitude of deposition and the affected regions varied substantially depending on the breathing phase and activity intensity. Table 3 summarizes the effects of different breathing conditions on aerosol deposition patterns within the human respiratory tract.

**Table 3:** Aerosol deposition trends as a function of breathing condition and flow rate

Reference	Breathing Condition	Flow Rate (L/min)	Affected Region	Deposition Trend	Key Findings
Kolanjiyil *et al*. [37]	Resting	15–30	ET-TB	Low-moderate	Relatively homogeneous deposition distribution
Calmet *et al*. [112]	Exercise	> 60	Nasal-ET	Very high	Significant increase in turbulence intensity
Ciloglu [87]	Heavy	≤ 60	ET	38% (7 µm)	Dominance of inertial effects

## The Effect of Aerosols

### Aerosol Size and Density

Aerodynamic diameter is a critical parameter governing aerosol transport, pulmonary penetration, and deposition within the airways. Taulbee and Yu [84] numerically investigated the distribution of particles in the 0.05–5 µm size range using Weibel’s A model and demonstrated that diffusion dominates for small particles, with minimum deposition occurring at a particle diameter of approximately 0.3 µm. While larger particles are generally retained in the ET region due to inertial effects, smaller particles in the 0.01–1 µm range can penetrate into the deeper lung regions. Robinson *et al*. [86] examined the behavior of cigarette smoke particles in TB airways using theoretical and experimental approaches and showed that submicron particles deposit on airway walls through convective diffusion during transport within the flow field. Zhang *et al*. [85] conducted the first comprehensive study of microscale particle transport under realistic inlet conditions, employing a TBU model to simulate local and total particle deposition in the TB region. The numerical findings were compared with experimental measurements and demonstrated strong consistency with analytical predictions.

Numerical studies performed on realistic MT models have demonstrated that total deposition of aerosols increases as particle size grows, with impaction being the dominant mechanism in ET region [28, 99]. The oral cavity acts as an efficient filter, accounting for a large fraction of total deposition, whereas deposition in the pharynx remains relatively limited. With an increase in airflow rate, larger particles deposit more prominently in the oral region, consistent with experimental observations [114]. Augusto *et al*. [115] investigated the deposition of 1–10 µm-sized particles under different breathing conditions using TBU models, showing that sedimentation dominates for larger particles at low velocities, while Brownian diffusion has only a limited influence and is relevant primarily for very small particles. Using nasal airway models from three individuals, Calmet *et al*. [116] analyzed the deposition of 2, 10, and 20 µm particles and demonstrated that both particle size and individual nasal geometry govern deposition, with 20 µm particles exhibiting high deposition and 2 µm particles showing lower and deeper penetration. Liu *et al*. [39] reported that in pediatric airways, deposition increases with particle density for particles larger than 0.5 µm, whereas for smaller particles this trend reverses as Brownian motion becomes dominant. Rahman *et al*. [117] similarly showed that high-density 10 µm particles exhibit higher deposition efficiency.

**Fig. 12:**
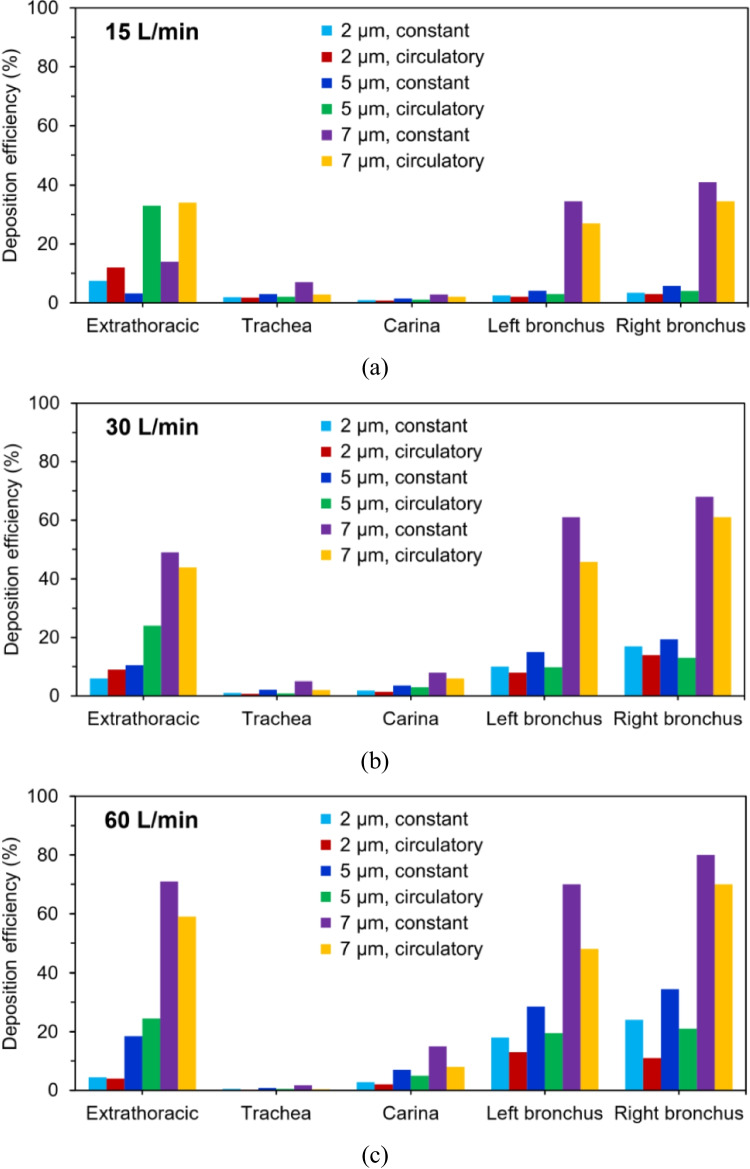
Particle deposition efficiencies in the airway model at different flow rates under steady and circulating respiration conditions; (**a**) 15 L/min, (**b**) 30 L/min, and (**c**) 60 L/min [21]

Kadota *et al*. [118] reported that in realistic airway models of patients with COPD, larger particles (5–10 µm) preferentially deposit in constricted bronchi (G2-G6), whereas smaller particles (1–2.5 µm) are retained in the bronchial tree with relatively low efficiency. In a CT-based realistic airway model, Ciloglu [21] demonstrated that cyclic breathing conditions lead to higher particle deposition in the bronchial region compared with steady breathing conditions. As airflow rate increases, smaller particles penetrate further into the bronchial region, while larger particles predominantly deposit in extrapulmonary regions. Figure 12 shows the size-resolved particle deposition fractions in different airway regions at inhalation flow rates of 15, 30, and 60 L/min, addressing how deposition varies with both particle size and breathing intensity.

### Morphology and Electrostatic Properties of Aerosols

Aerosol particle shape and morphology significantly affect their behavior in terms of transport and deposition in the human respiratory tract. Computational studies often approximate non-spherical particles as clusters of overlapping spheres, an approach widely used in simplified bifurcation models [119]. Using a realistic MT model, Jia *et al*. [120] investigated the deposition of microparticles with different shapes and demonstrated that deposition efficiency decreases as particles deviate from sphericity, due to a reduction in drag force. Similarly, Shachar-Berman *et al*. [121] reported that fibrous particles with high aspect ratios exhibit a higher deposition potential in the bronchiolar and acinar regions. Feng and Kleinstreuer [122] further showed that high–aspect-ratio particles tend to penetrate more deeply into the lung. In addition, it has been reported that deposition of non-spherical particles, similar to spherical ones, increases with increasing airflow rate.

Particle charge also plays a significant role in aerosol deposition. Piemjaiswang *et al*. [123] demonstrated that charged particles migrate from the core flow toward the airway walls due to electrostatic effects. These findings were supported by *in vitro* experiments performed by Azhdarzadeh *et al*. [124], which showed a marked increase in deposition for charged particles smaller than 3 µm. Using CFD-DEM simulations, Piemjaiswang *et al*. [125] examined the deposition behavior of drug particles of varying shapes, sizes, and densities and found that tomahawk-shaped particles exhibited the highest deposition efficiency. While increases in particle size and density enhanced deposition via impaction, shape-size interactions produced distinct behaviors, particularly for small particles, with deposition being highest in G0 and lowest in G3.

### Particle Injection Position and Duration

**Fig. 13:**
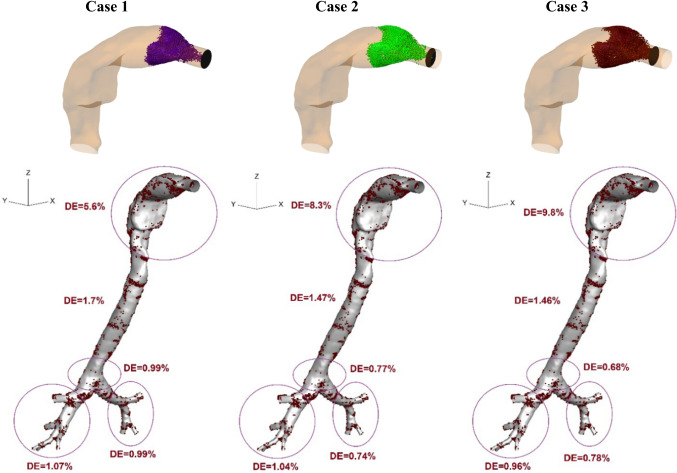
Deposition efficiency in various parts of the human respiratory system under vortex-free flow with particle injection in three different planes [126]

Numerous studies have demonstrated that the location of particle injection significantly influences deposition patterns, pointing out that targeted delivery to the particular regions of the lungs can be achieved through controlled injection positioning. Taheri *et al*. [126] studied the deposition efficiency of 2 µm particles released from different oral injection locations and injection diameters under normal breathing conditions (30 L/min) in a realistic human airway model (Fig. 13). Their results showed that releasing particles deeper within the oral cavity or randomly injecting them with a smaller injection diameter (d_inj_​) under vortical flow conditions reduced drug delivery efficiency by 50–80%. The authors emphasized that airflow conditions are a critical parameter for reliable drug delivery, and that maintaining a uniform inlet flow while avoiding vortical structures is more beneficial for efficient aerosol transport.

Using a WLAM model, Kolanjiyil and Kleinstreuer [58] identified optimal drug release locations and particle emission maps by backward tracking of particle trajectories. Wang *et al*. [127] examined the delivery of drugs to targeted lung regions in a TB model designed for COVID-19 patients and found that specifying the particle release locations at the inlet substantially improved the fraction of particles reaching the intended targets. Similarly, Wang *et al*. [128] defined an optimized release map that maximized particle deposition in a TB model of an 11-year-old child with bronchopneumonia. Other studies investigating the correlation between injection positioning and intrapulmonary deposition patterns have also confirmed that injection timing strongly influences particle transport and deposition processes [129]. In addition, it has been reported that increasing injection duration leads to reduced particle deposition rates, specifically, extending the injection duration from 1 to 4 s resulted in deposition reductions of 60% and 33% in USP and MT models, respectively [10].

### Hygroscopic Growth, Evaporation, and Phase-Change Dynamics

Hygroscopic particle growth occurs as a function of relative humidity and water vapor levels within the respiratory tract and can substantially influence aerosol deposition by increasing particle inertia and enhancing impaction mechanisms. In the upper airways, relative humidity has been shown to exceed 100% under saturated airflow conditions, under which particle size may increase by more than a factor of two [57, 130]. In simplified airway models, hygroscopic growth under wet conditions has been reported to increase deposition efficiency compared with dry conditions; however, latent heat loss associated with mucus evaporation can limit this growth and reduce deposition by up to 10% [131]. Moreover, neglecting hygroscopic effects in G3-G6 airways has been shown to lead to substantial underestimation of aerosol deposition [132].

Nanoparticles typically traverse the MT region with low deposition and are largely exhaled; however, hygroscopic growth enhances their retention in the deep airways. Based on this principle, the Enhanced Condensational Growth (ECG) approach markedly increases nanoparticle deposition in airway generations G0-G5 compared with conventional conditions [133].

Heat and mass transfer processes within the mucus-tissue layer play a crucial role in controlling the behavior of evaporating aerosols [47]. Multiple studies have confirmed that environmental temperature, humidity, and inlet conditions play decisive roles in particle growth and deposition [134, 135]. Analyses under cyclic inspiration indicate that droplet deposition is largely dependent on initial particle size, with the 5–10 µm range representing a critical size interval that is particularly sensitive to relative humidity [136].

In addition to hygroscopic growth driven by moisture uptake, phase-change processes such as evaporation, condensation, and droplet shrinkage also play a critical role in determining aerosol transport and deposition within the respiratory tract. In spray-based delivery systems, including metered-dose inhalers (MDIs) and nebulizers, droplets may undergo rapid solvent evaporation immediately after actuation, leading to substantial reductions in particle diameter and corresponding changes in aerodynamic behavior [131]. Since deposition mechanisms are highly size-dependent, such transient variations can significantly alter regional and total deposition patterns. Several CFD studies have therefore incorporated coupled heat- and mass-transfer models to account for droplet evaporation and condensation dynamics, demonstrating that neglecting these effects may result in inaccurate prediction of particle trajectories and delivered dose [47, 132]. Consequently, realistic representation of aerosol phase-change phenomena is essential for reliable simulation of inhalation drug delivery.

### Mucus Layer Effects on Aerosol Deposition and Post-Deposition Fate

The airway mucus layer is not merely a passive coating but a dynamic, viscoelastic barrier that governs near-wall transport, particle capture, and post-deposition fate. Lining the conducting airways as part of the airway surface liquid, the mucus layer traps particles upon wall contact and introduces additional time scales associated with diffusion and penetration across the mucosal barrier, as well as removal via cilia-driven mucociliary clearance (MCC) [137]. Accordingly, many CFD-based aerosol transport studies represent mucus effects using fully trapping (“100% sticky”) wall boundary conditions, reflecting the adhesive nature of the mucus layer and its filtration function in the conducting airways [49]. More advanced numerical frameworks have incorporated mucus-induced stickiness, soft-wall assumptions, or explicit representations of the mucus and periciliary layers to predict near-wall transport and particle penetration through the mucosal barrier [49, 138].

Beyond initial deposition, recent CFD, multiphase, and experimental studies demonstrate that the mucus layer critically modulates post-deposition particle fate, including evaporation dynamics, penetration, residence time, and ultimately drug retention. Explicit representation of mucus-related heat and mass transfer has been shown to substantially suppress droplet evaporation, by up to ~ 90% for initially 10–12 μm droplets, thereby altering delivered size distributions and penetration behavior, even when changes in total deposition fraction remain limited [136]. While deposition is largely controlled by particle size, inertia, and airway geometry, mucus properties strongly influence retention and clearance, particularly for particles in the critical 5–10 μm size range [139]. Mechanistic models incorporating mucociliary transport provide an essential link between deposition location and residence time, helping to explain how poorly soluble or strongly mucus-binding particles may be rapidly removed under normal conditions, yet exhibit enhanced retention in diseases characterized by mucus hypersecretion and impaired clearance, such as asthma, COPD, or cystic fibrosis [140–142]. These findings underscore mucus as a key determinant of post-deposition aerosol dynamics and highlight the importance of coupling deposition predictions with mucus transport physics for accurate inhalation dosimetry.

## Future Research Directions and Emerging Challenges

### Multiscale and Physiologically Realistic Modeling Frameworks

Despite substantial progress in CFD-based modeling of respiratory airflow and aerosol transport, several challenges remain that must be addressed to further improve predictive accuracy, physiological realism, and clinical applicability. A key direction is the development of multiscale and hybrid modeling frameworks that combine turbulence-resolving approaches in the upper airways with RANS or laminar formulations in the distal regions, enabling whole-lung simulations while maintaining computational feasibility. Such strategies are expected to provide a more balanced compromise between fidelity and cost, particularly for repeated breathing cycles and large parametric studies.

Another major challenge concerns intra- and intersubject airway variability, which often dominates deposition outcomes yet remains difficult to represent systematically. Future efforts should therefore focus on population-based modeling, patient-specific reconstructions, and uncertainty quantification techniques, including probabilistic simulations and sensitivity analyses, to better characterize the variability and robustness of predicted deposition patterns. These approaches will be essential for improving the reliability and translational relevance of respiratory CFD studies.

In addition, emerging data-driven and multiphysics approaches offer promising opportunities for the next generation of respiratory simulations. Machine learning–based surrogate models, reduced-order methods, and physics-informed frameworks may significantly accelerate computations and enable inverse design of inhalation strategies, while moving-wall and fluid–structure interaction formulations can provide more realistic representations of lung deformation and distal airflow behavior. Together, these advances are expected to further enhance the predictive capability and practical impact of CFD-based respiratory modeling.

### Uncertainty Quantification and Sensitivity Analysis

Most computational studies of respiratory airflow and aerosol deposition have traditionally adopted deterministic formulations, where model inputs such as breathing profiles, particle properties, and airway geometries are treated as fixed quantities. However, in real physiological conditions, these parameters exhibit substantial inter- and intra-subject variability, which may lead to significant uncertainty in predicted deposition outcomes. Consequently, uncertainty quantification (UQ) and sensitivity analysis are increasingly recognized as essential components of next-generation respiratory CFD modeling.

Stochastic approaches, including Monte Carlo sampling, probabilistic particle size distributions, and variability-aware breathing or geometry models, enable estimation of confidence intervals rather than single deterministic predictions. In parallel, sensitivity analyses help identify the dominant parameters governing regional and total deposition, thereby guiding model simplification and experimental prioritization. Incorporating these techniques can substantially enhance the robustness, clinical relevance, and predictive reliability of inhalation drug delivery simulations, representing an important direction for future research.

### Artificial Intelligence (AI) and Data-Driven Modeling in Respiratory CFD

Recent advances in machine learning (ML) and AI have opened new avenues for accelerating respiratory flow and aerosol transport simulations, addressing key limitations of conventional CFD approaches. Physics-informed neural networks (PINNs), which embed governing equations such as the Navier–Stokes system into learning frameworks, have demonstrated comparable accuracy to high-fidelity CFD while reducing mesh sensitivity and offering flexibility in dealing with sparse data and patient-specific anatomies. Applications in respiratory modelling include PINN-based airway flow reconstruction and surrogate predictions across varying breathing conditions, which can substantially reduce computational cost relative to traditional solvers. Beyond PINNs, generative and operator-learning approaches such as DeepONets and Fourier Neural Operators hold promise for mapping complex parametric spaces of airway geometry and breathing profiles, enabling ultra-fast predictions once trained. These data-driven tools can also be integrated with imaging pipelines (e.g., 4D CT/MRI) to facilitate real-time or near-real-time simulation of airflow and particle dynamics in patient-specific models. However, challenges remain in ensuring generalizability across diverse physiological conditions, enforcing accurate boundary conditions within learned models, and establishing standardized benchmarks for validation against experimental or clinical data [143].

## Conclusions

In this study, the principal modeling approaches used in the literature for modeling airflow and aerosol particle dynamics within the human respiratory system were examined from a systematic and critical perspective. The review reveals that respiratory airway models differ substantially in terms of geometric representation, computational cost, and the physical mechanisms they are able to resolve. One-dimensional and idealized models, especially, while useful for understanding average transport trends, are insufficient for capturing local flow structures and heterogeneous particle deposition. In contrast, CT-based 3D realistic geometries enable more accurate prediction of turbulent flow features and localized deposition patterns, especially in the upper airway region.

The analysis further shows that turbulence model selection plays a decisive role in respiratory CFD simulations. Many studies rely predominantly on RANS-based formulations without sufficient justification, whereas the literature indicates that LES is more suitable for resolving post-glottal jets, flow separation, and transient vortical structures in the extrathoracic region. By comparison, laminar or k–ω-based RANS models provide an appropriate balance between accuracy and computational cost in the tracheobronchial and alveolar regions. The comparisons and decision frameworks presented in this review highlight the necessity of region-specific, physics-informed modeling strategies for reliable prediction of airflow and aerosol transport.

Continued advances in multiscale modeling, realistic airway representations, and integrated computational strategies are expected to substantially enhance the predictive capability, robustness, and translational value of CFD-based respiratory simulations for both pharmaceutical and environmental health applications.

## Data Availability

The data supporting the findings of this study are available from the corresponding author upon reasonable request.
